# Combined Effects of Flow Diverting Strategies and Parent Artery Curvature on Aneurysmal Hemodynamics: A CFD Study

**DOI:** 10.1371/journal.pone.0138648

**Published:** 2015-09-23

**Authors:** Jinyu Xu, Zhichen Wu, Ying Yu, Nan Lv, Shengzhang Wang, Christof Karmonik, Jian-Min Liu, Qinghai Huang

**Affiliations:** 1 Department of Neurosurgery, Changhai Hospital, Second Military Medical University, Shanghai, China; 2 School of International Relations and Public Affairs, Fudan University, Shanghai, China; 3 Department of Mechanics and Engineering Science, Fudan University, Shanghai, China; 4 Cerebrovascular Center, Department of Neurosurgery, Houston Methodist Hospital, Houston, TX, United States of America; Technion - Israel Institute of Technology, ISRAEL

## Abstract

**Purpose:**

Flow diverters (FD) are increasingly being considered for treating large or giant wide-neck aneurysms. Clinical outcome is highly variable and depends on the type of aneurysm, the flow diverting device and treatment strategies. The objective of this study was to analyze the effect of different flow diverting strategies together with parent artery curvature variations on altering intra-aneurysmal hemodynamics.

**Methods:**

Four ideal intracranial aneurysm models with different parent artery curvature were constructed. Computational fluid dynamics (CFD) simulations of the hemodynamics before and after applying five types of flow diverting strategies (single FD, single FD with 5% and 10% packing density of coils, two FDs with 25% and 50% overlapping rate) were performed. Changes in pressure, wall shear stress (WSS), relative residence time (RRT), inflow velocity and inflow volume rate were calculated and compared.

**Results:**

Each flow diverting strategy resulted in enhancement of RRT and reduction of normalized mean WSS, inflow volume rate and inflow velocity in various levels. Among them, 50% overlapped FD induced most effective hemodynamic changes in RRT and inflow volume rate. The mean pressure only slightly decreased after treatment. Regardless of the kind of implantation of FD, the mean pressure, inflow volume rate and inflow velocity increased and the RRT decreased as the curvature of the parent artery increased.

**Conclusions:**

Of all flow diverting strategies, overlapping FDs induced most favorable hemodynamic changes. Hemodynamics alterations post treatment were substantially influenced by parent artery curvature. Our results indicate the need of an individualized flow diverting strategy that is tailored for a specific aneurysm.

## Introduction

Endovascular treatment for intracranial aneurysms (IAs) has been developed over the past decade as an alternative to surgery. Stent-alone treatment, as one of the endovascular technique, allows not only for protection of the parent artery from occlusion, but also diversion of the blood flow from the aneurysm [1–4]. In order to enhance the diverting effect, the flow diverter (FD) device that is stent-like but with much higher metal coverage, has emerged in recent years. Its effectiveness in treating aneurysms had been demonstrated in both animal and clinical studies [5,6], especially for wide-necked, large or fusiform aneurysms [7–9].

Although FD is being increasingly deployed to treat cerebral aneurysms, clinical outcomes are still highly variable. Besides complete aneurysm occlusions in most cases, longer-term patency and delayed aneurysm rupture have been reported [10–13]. The FD does not act by only mechanically excluding the aneurysm from blood flow but—depending on the induced hemodynamic changes—initiates a process of thrombosis formation and endothelial remodeling to eventually seal the aneurysm. Hence the prognosis of FD-treated aneurysms will be mainly affected by changes in hemodynamics. Many factors may influence aneurysmal hemodynamics, such as variation in aneurysms geometry and the properties of FD itself. Differences in wire density had been studied using idealized geometries by Seshadhri et al. [14] and the FD with the highest wire density was shown to induce the most significant hemodynamic change. However, it is still not fully understood whether the variations in clinical outcome are related to different morphologies of the parent artery in particular its curvature, or the variable types of the flow diverting device and the particular treatment strategy. This study we designed to address the following questions:

1. How should we use the flow diverters for treating aneurysms? Single FD, multiple FDs or FD combined with loose-packing coils?
2. Should we plan the FD treatment based on patient specific vessel geometry, such as different parent vessel curvatures?

## Materials and Methods

The Institution Review Board of Changhai Hospital, affiliated to the Second Military Medical University, approved this retrospective study, and the requirement for informed consent was waived. In addition, we have not conducted research outside our country of residence.

### Vascular Models

**Fig 1:**
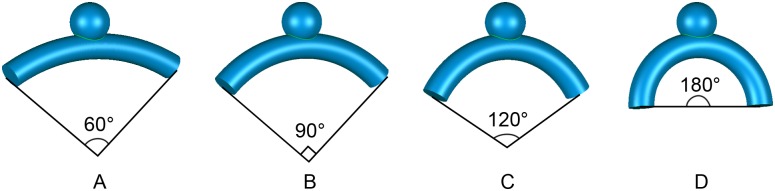
Four ideal intracranial aneurysm models with different parent vessels' curvature. A, 60°. B, 90°. C, 120°. D, 180°.

Four ideal intracranial aneurysm models with wide necks that were usually considered for flow diverting treatments were constructed using 3DMAX8.0 (Autodesk USA). The perpendicular height, neck diameter and aspect ratio of these aneurysm models were 5 mm, 5 mm and 1, respectively. The length and diameter of their parent vessel was 30 mm and 4 mm. The curvatures of the parent vessel segments were 34.89, 52.33, 69.78, 104.67 m^-1^, respectively (corresponding to the angles of the arc: 60°, 90°, 120° and 180°) (Fig 1).

**Fig 2:**
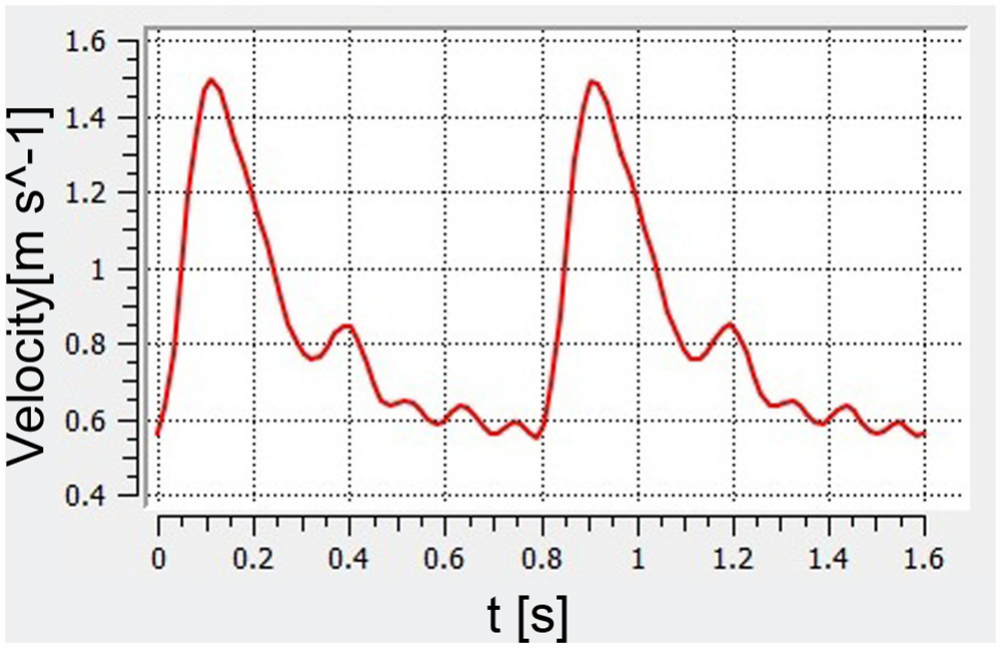
The flow velocity waveform based on transthoracic duplex Doppler examination.

From a retrospectively obtained pulsatile velocity waveform recorded with transcranial Doppler of a healthy subject, an average blood flow velocity waveform was calculated (flow spectrum envelope using Matlab 7.0 software, MathWorks, Natick, Massachusetts) (Fig 2). The maximum velocity magnitude was 1.49 m/s. Plug flow was used for the inlet and care was taken to ensure that the proximal section of the parent artery was longer than 10 artery diameters so that the flow profile could fully develop prior to the aneurysm ostium.

### Virtual FD Treatment Models

Five different types of FD treatment strategies were simulated based on three common clinical practices: 1. single FD; 2. single FD assisted with coil embolization (5% packing density); 3. single FD assisted with coil embolization (10% packing density); 4. two overlapping FDs with 50% of overlapped rate at the aneurysm neck; and 5. two overlapping FDs with 25% of overlapped rate.

Virtual FD models were constructed (SolidWorks 2010, Dassault Systemes, Concord, Massachusetts) according to the design specifications of the FD (first-generation of the TUBRIDGE embolization device composed of 32 nickel–titanium alloy struts, including two parallel radio-opaque struts containing platinum (diameter = 0.05 mm), MicroPort Medical [Shanghai] Co. Ltd., China), which included the FD's inner/outer diameter, filament diameter, number of filaments and pitch as previously described [15]. The diameter of the FD was adjusted to match the diameter of the parent artery. The virtual FD models were bent (Abaqus 6.11, SIMULIA, Providence, Rhode Island) to follow each parent vessels’ curvature (uniquely defined by the angle of the ideal arc geometry) and stent struts not covering the ostium were removed GEOMAGIC STUDIO 9.0 (Geomagic USA). The metal coverage of each FD was 30%.

The endosaccular deployment of the platinum coils were modeled as porous medium as described by Mitsos et al. [16]. All coils had a diameter of 0.010 inch (i.e. 0.25 mm). The packing densities were chosen as 5% and 10%, which corresponds to a porosity of 0.95 and 0.90, respectively. Corresponding permeability was calculated from taking aneurysm volume and porosity into account (Table 1). The corresponding drag factor in the porous medium was estimated to CD ≈2.2 [17].

**Table 1:** Permeability of Each Aneurysm Models.

Aneurysm models	Volume of Aneurysm (cm^3^)	Coil Diameter (mm)	Coil porosity	Coil permeability (m^2^×10^−7^)
60°	0.11536	0.25	0.95	6.89891
	0.11536	0.25	0.90	5.86593
90°	0.11487	0.25	0.95	6.89884
	0.11487	0.25	0.90	5.86588
120°	0.11472	0.25	0.95	6.89882
	0.11472	0.25	0.90	5.86586
180°	0.11335	0.25	0.95	6.89863
	0.11335	0.25	0.90	5.86570

### CFD Meshing and Flow Modeling

**Fig 3:**
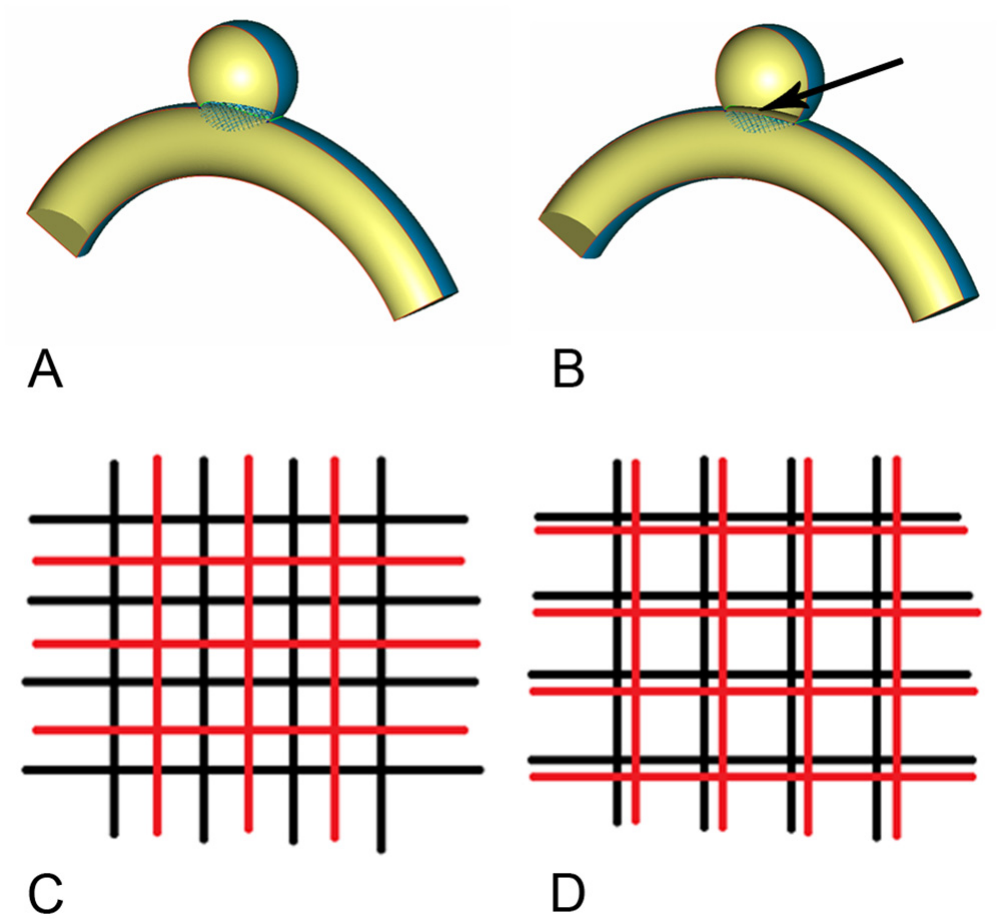
The geometry of models. A, the region of the FD model. B, the region of the FD and porous medium for coil, the interface (arrow) of the fluid (vessel region) and porous medium (aneurysm region). C, 50% overlapped rate of double FD models. D, 25% overlapped rate of double FD models.

The advantage of using ideal geometries allows the unique definition of the aneurysm ostium and the constant curvature provides an unambiguous approach of uniquely fitting the virtual FD models into the parent artery segment. The position of the implanted FD, of the porous medium representing the coils and of the two different overlapping FD configurations are shown in Fig 3. Each model was meshed (ICEM CFD 11.0, ANSYS, Canonsburg, PA) with the same reference mesh size to generate 1.01 to 1.72 million finite volume tetrahedral elements and wall prism elements (for accurate boundary layer resolution), with 2.03×10^3^ to 3.51×10^3^ elements per cubic millimeter [15]. Previous studies have shown that this mesh resolution is sufficient for hemodynamic simulations [18].

The governing equations underlying the CFD computation were the Navier-Stokes equations assuming laminar and incompressible flow. Blood was treated as a Newtonian fluid. Density and dynamic viscosity of blood were specified as ρ = 1050 kg/m^3^ and μ = 0.00345 Pa·s, respectively. Vessel walls were assumed to be rigid with no-slip boundary conditions. On the inlet, the measured pulsatile velocity waveform obtained as described above was prescribed. The outlet was modeled as an open boundary with zero static reference pressure. The simulation was performed by CFX 11.0 (ANSYS, Canonsburg, PA). We discretized the whole cardiac cycle of 0.8 s by a time-step of 0.001 s. Three cardiac cycles were simulated to allow for the decay of initial transients, and results are reported from the last cycle only. The aneurysm geometries were isolated from their parent arteries for subsequent data analysis by a cut plane positioned at the ostium. We then post-processed and visualized the results of these simulations with CFX 11.0 (ANSYS, Canonsburg, PA).

### Hemodynamic Parameters

Changes of the following hemodynamic parameters were calculated before and after the five types of flow diverting strategies: pressure, wall shear stress (WSS), relative residence time (RRT), inflow velocity and inflow volume rate. WSS (already time-averaged, as in Eq 1) was averaged over the sac area (the entire luminal surface of the aneurysm sac). In this study, the WSS distributions were normalized by the average parent vessel WSS in the same model to allow comparison among different models [19]. And as we assume that our models accurately reproduce 'healthy' or 'normal' WSS values in the parent vessel, the deviations from these values are best described by providing relative values (relative to these normal values). RRT, a combination of WSS and Oscillatory Shear Index (OSI), describes the slow flow adjacent to the aneurysm wall by providing a means of quantifying the residence time near the wall [20]. Thus, a metric termed RRT was defined to quantify the state of disturbed flow [21]:

$$WSS=\frac{1}{T}\int_{0}^{T}\left|wss_{i}\right|dt$$ (1)

$$OSI=\frac{1}{2}\left\{1-\frac{\left|\int_{0}^{T}wss_{i}dt\right|}{\int_{0}^{T}\left|wss_{i}\right|dt}\right\}$$ (2)

$$RRT=\frac{1}{(1-2\times OSI)\times WSS}=\frac{1}{\frac{1}{T}\left|\int_{0}^{T}wss_{i}dt\right|}$$ (3)

where wss_i_ is the instantaneous WSS vector and T is the duration of the cycle.

The mean inflow velocity and inflow volume rate were calculated at the aneurysm neck at peak systole (t = 0.11s). For this, the flow rate (separately for inflow and outflow) through the cut plane separating the aneurysm from the parent artery at the location of the ostium was calculated as the product of the velocity component normal to the area and the area (built-in function available in CFX 11.0, ANSYS, Canonsburg, PA). We also visualized the inflow stream into the aneurysm sac on a plane oriented perpendicular to the aneurysm ostium before and after treatments.

## Results

**Fig 4:**
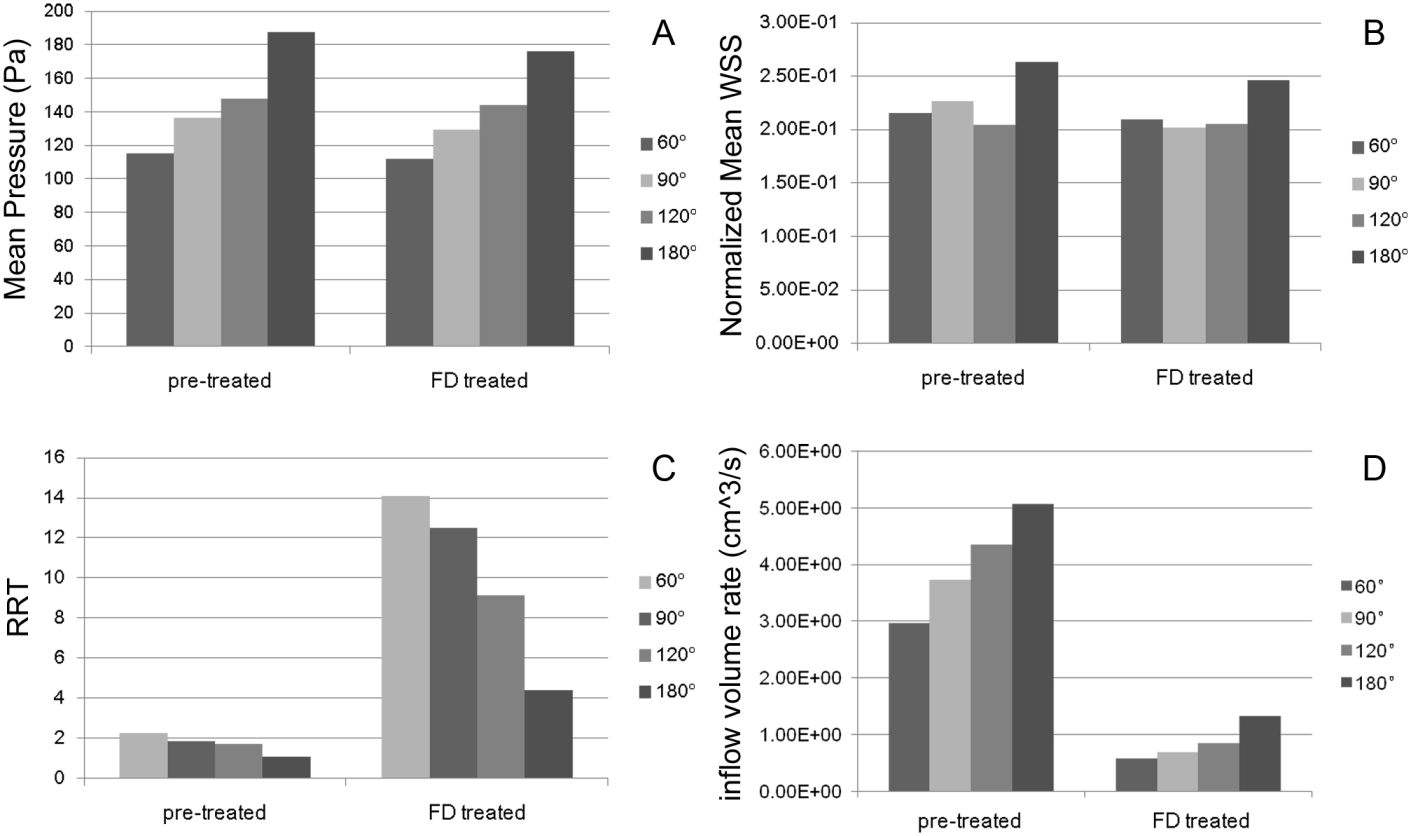
Quantitative hemodynamic results before and after FD treatment. Each panel shows the hemodynamic results for every parent vessels' curvature considered (first, second, third and forth bars indicate 60°, 90°, 120° and 180° respectively). A, mean pressure. B, normalized mean WSS. C, RRT. D, inflow volume rate.

As the parent vessels' angle increases from 60°, 90°, 120° to 180°, the mean pressure and inflow volume rate increased and the RRT decreased (Fig 4). The mean pressure increased from 115.26, 136.49, 148.05 to 187.65 Pa, the inflow volume rate increased from 2.96, 3.73, 4.35 to 5.06 cm^3^/s, and the RRT decreased from 2.28, 1.84, 1.72 to 1.09. This tendency, however, was not obvious in the normalized mean WSS with the normalized mean WSS for 60° being smaller than the one for 180° (0.22 versus 0.26).

After single FD treatment, a similar trend in hemodynamic changes depending on parent artery curvature was observed. As the parent vessels' angle increased from 60°, 90°, 120° to 180°, the mean pressure grew from 111.72, 129.49, 143.88 to 175.99 Pa, the inflow volume rate also showed an upward trend from 0.58, 0.69, 0.85 to 1.33 cm^3^/s, and the RRT decreased from 14.09, 12.50, 9.15 to 4.39. Though the tendency of the normalized mean WSS was not as obvious as other hemodynamic parameters, the normalized mean WSS of 60° was still smaller than that of 180° (0.21 versus 0.25).

**Fig 5:**
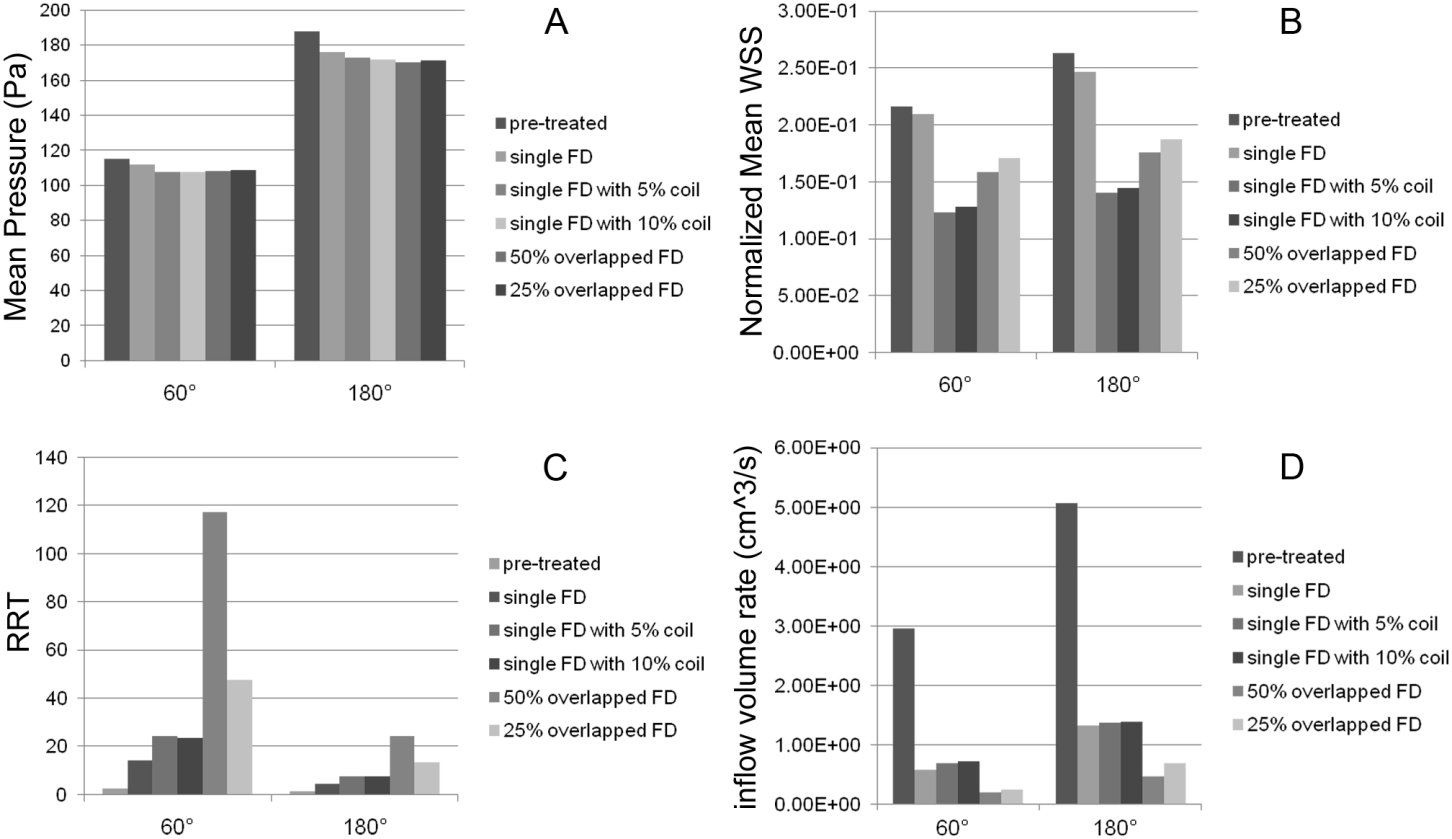
Quantitative hemodynamic results before and after each types of flow diverting methods. The two panels show the hemodynamic results for 60° and 180° parent vessels' curvature (first to sixth bars indicate pre-treated, single FD, single FD with 5% packing density coil, single FD with 10% packing density coil, overlapped FD with 50% overlapped rate and overlapped FD with 25% overlapped rate respectively). A, mean pressure. B, normalized mean WSS. C, RRT. D, inflow volume rate.

The influence of different FD treatment strategies on aneurysm hemodynamics was demonstrated in Fig 5. The mean pressure only slightly decreased after treatments, and no significant differences were observed for the different FD treatment strategies. The RRT was enhanced, and the normalized mean WSS and inflow volume rate were reduced. Specifically, the reduction of normalized mean WSS was largest for the single FD combined with 5% coils, and smallest using only the single FD. The treatment using overlapped FD with 50% overlapped rate resulted in the most significant enhancement of RRT and reduction of inflow volume rate, while the single FD only showed the lowest enhancement of RRT. Combining single FD with 10% packing density coil showed the lowest reduction of inflow volume rate. There was no significant hemodynamic difference between the FD treatments combined with 5% and 10% packing density coil. There was also no significant difference in the change of normalized mean WSS and inflow volume rate between the two degrees of overlapping FDs, but significant differences were found for the increase of RRT.

**Fig 6:**
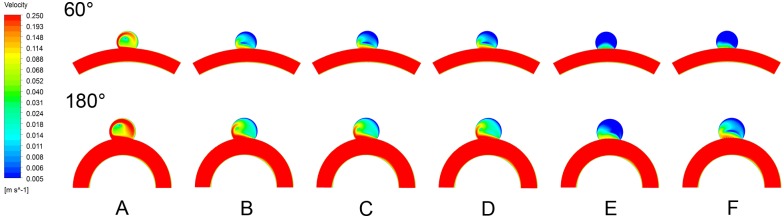
The velocity magnitudes of inflow stream of the aneurysm sac was plotted in logarithmic scale on the cut plane before and after each types of flow diverting methods for 60° and 180° parent vessels' curvature considered. A, pre-treated. B, single FD. C, single FD with 5% packing density coil. D, single FD with 10% packing density coil. E, overlapped FD with 50% overlapped rate. F, overlapped FD with 25% overlapped rate.

The inflow velocity was substantially reduced after flow diverting treatment as shown (Fig 6) indicating that the FDs blocked and disrupted the inflow jets thereby reducing aneurysmal inflow. Among all five FD treatment strategies, the method of overlapping FD, in particular with a degree of 50% overlap, showed the most significant reduction in inflow velocity. The inflow jet into the aneurysm exhibited a consistent upward trend both before and after flow diverting treatments as the parent vessel curvature increased (Fig 6).

## Discussion

In this study, we analyzed hemodynamic changes caused by different flow diverting treatment strategies together with varying degrees of parent artery curvature. All kinds of flow diverting strategies investigated resulted in enhancement of RRT and reduction of normalized mean WSS, inflow volume rate and inflow velocity. Except for the mean pressure, different types of flow diverting strategies led to different levels of hemodynamic changes. Moreover, the pressure, inflow velocity and inflow volume rate increased and the RRT decreased as the parent vessels' curvature increased, regardless of the way the FD was implanted.

Hemodynamic factors are commonly believed to play an important role in the pathogenesis, progression, and rupture of cerebral aneurysms. Specifically the hemodynamic-change-induced intra-aneurysmal thrombosis and thrombus are the most important processes after the endovascular treatment for healing, and the low-flow velocity relates to regions prone to thrombus formation [22]. These factors were supported by our previous study [23] that the higher RRT increment, more percentage of inflow volume reduction and location of stream inlet near the central part of the neck may be closely related to healing. It was also demonstrated that relative flow velocity and WSS reduction caused by FD implantation resulted in aneurysm thrombosis in a majority of cases [3]. Significant correlations between regions where CFD predicted either an increased flow residence time (RT) or low WSS and the regions where thrombus deposition was observed to occur using MRI in vivo has been previously demonstrated [24]. Reduction of blood flow into the aneurysm and of the flow velocity magnitude at the neck were found to be related to favorable outcome for FD treatment [4]. These results all imply the importance of hemodynamics for FD treatment outcome.

CFD studies using real patient geometries depend on many factors including parent artery curvature, aneurysm morphology. Using ideal geometries allows isolating the effect a single factor in this multifactorial space has on the overall hemodynamics. The boundary conditions we used are intended to represent the pressure drop across the artery segment containing the aneurysm. Inlet pressure is defined by the inlet velocity and outlet pressure is set to zero (as baseline) [3,4,10,18,23,25–30]. This approach of investigating the effects of the pressure gradient on a local artery segment is a commonly used and an accepted approach and therefore allows our results to be compared directly to simulations results reported in the literature obtained in this fashion [3,4,10,18,23,25–30].

Most previous studies were based on virtual stenting methods, including but not limited to the adaptive grid embedding technique, porous medium method, fast virtual stenting method [30–32]. Ma et al. [33] simulated the mechanical deployment of FDs in patient-specific aneurysms by using a finite element analysis (FEA) based workflow. Our previous study compared the real structural configurations of fully deployed FDs in vivo with those of virtual FDs, and demonstrated that both virtual and realistic FD deployment methods produced very similar hemodynamic results, with metal coverage (MC) in-vivo being 24.97% (range 21.19 to 29.45%) and 30% for the simulations [15].

The hemodynamic efficiency of a stent or FD is related to several parameters, including strut shapes, porosity, quantity of stents, mesh hole shapes, metal coverage (MC) [34–36]. Liou et al. previously reported that the total inflow to the aneurysm is reduced to approximately 75%, 37.5% and 25% for the one-, two- and three-layer stented models, respectively, in comparison to an unstented model [37]. These findings are in agreement with our results when using two different degrees of FD overlap. Increase in RRT and reduction of normalized mean WSS, inflow volume rate and inflow velocity, were much more significant in the case with overlapping FDs than with single FD suggesting that the method of overlapping FDs, especially with a degree of 50%, tend to generate more favorable hemodynamic changes: Inflow at highest curvature (180 degrees) is reduced to a third compared to using a single FD, which is about the same factor as for the lowest curvature (60 degrees). At the same time, the total inflow at the highest curvature is still a factor 2.3 higher with 50% overlap than at the lowest curvature. These findings indicate the use of more than one FD is advisable only at high curvatures (> 60 degrees); at lower curvatures, risks for potential technical complications during the procedure and increased risk for thrombus formation in the parent artery may not outweigh the benefits of the achieved inflow reduction. Keeping these potential complications in mind, our results suggest that a higher and constant pore density over the entire length of the aneurysm neck leads indeed to a more efficient flow diversion and durable aneurysm occlusion [38]. Higher pore density can be achieved by more metal coverage. More struts may mean less flexibility of the final construct making a good fit into a tortuous curved arteries challenging. A new generation Tubridge FD device is being designed increasing the number of struts from 32 to 64 with the same material property for better pore density. We limited our new Tubridge FD to a maximum of 64 struts after carefully testing its flexibility and evaluating the probability of blocking or modifying the blood supply of small branches in recent clinical applications [39]. Covered stents are not used in the cerebral vasculature as they might block off small side branches and perforators causing stroke. The generality of our findings may be transferrable to other kinds of flow diverter devices with different size, shape, number of struts as inflow into the aneurysm pre and post FD treatment is mainly governed by the geometry of the parent vessel.

Hemodynamic changes can be affected not only by the FD deployment but also by the morphology of the aneurysm and the parent artery, as the morphologies of the parent artery and of the aneurysm are as important as hemodynamics in discriminating aneurysm rupture status [40,41]. Cebral et al. [42] showed that hemodynamics with a given geometry did not vary significantly with physiological variations of flow rate, blood pressure, and waveform. It can be inferred that intra-aneurysmal hemodynamics might be predominantly dependent on the geometry of the aneurysmal sac and its parent vessel [41,43,44]. Szikora et al. [45] noted that the aneurysm-to-parent-vessel angle was the most significant determinant of flow patterns in the sac. Our results also show a strong dependence of aneurysm hemodynamic on the curvature of the parent artery. Specifically, pressure, inflow volume rate and inflow velocity increased and the RRT decreased as the curvature increased.

### Limitations

1. Although CFD allows simulating the intra-aneurysmal hemodynamic environment with potential validation by clinical data, it has inherent limitations. The biological process of aneurysm prognosis, which is also related to the function of platelets, the local thrombosis, aneurysm occlusion, is not been taken into account in the kind of CFD simulation performed here.
2. While modeling coils as porous media is easy to implement, it cannot account for the actual coil locations and for local differences in permeability (as employed here).
3. Due to the lack of vessel wall thickness and elasticity parameters for intracranial aneurysm, vessel walls were assumed rigid for this study. A more realistic approach may employ deformable vessel walls or fluid-solid coupling and should be used one accurate material properties for the aneurysm wall become available.
4. To make the simulations more realistic, patient-specific inflow and outflow boundary conditions should be based on measured values [46,47].
5. Finally, in order to compare the hemodynamic differences among aneurysm models with different vascular morphology, our study adopted ideal intracranial aneurysm models which only changed its parent vessels' curvature within 2D plane, not considering the influence of its parent vessels' 3D torsion.

## Conclusion

In this study, we analyzed hemodynamic changes induced by different flow diverting strategies together with variation in the curvature of the parent artery. FDs overlapping by 50%, induced most favorable hemodynamic changes. Intra-aneurysmal flow was substantially influenced by the curvature of the parent artery indicating the, need for an individualized flow diverting treatment strategy, such as the number of FD needed (perhaps by 'stacking' the FD or by using two overlapping devices) or the inclusion of coils with specific packing density, for a particular aneurysm.

## Supporting Information

S1 Table Quantitative hemodynamic results before and after each types of flow diverting methods for each vessels’ curvature. (PDF)
