# Supplementary material for: Combined Effects of Flow Diverting Strategies and Parent Artery Curvature on Aneurysmal Hemodynamics: A CFD Study
Source: PLoS One. 2015 Sep 23;10(9):e0138648. doi: 10.1371/journal.pone.0138648 (PMC4580450; doi:10.1371/journal.pone.0138648)
Supplement: S1 Table — (PDF) [file pone.0138648.s001.pdf]

S1 Table. Quantitative hemodynamic results before and after each types of flow diverting methods for each vessels' curvature

	flow diverting methods	mean pressure (Pa)	normalized mean WSS	RRT	inflow volume rate (cm^3/s)
60°	pre-treated	115.26	0.22	2.28	2.96
	single FD	111.72	0.21	14.09	0.58
	overlapped FD with 50% overlapped rate	108.16	0.16	117.16	0.20
	overlapped FD with 25% overlapped rate	108.98	0.17	47.57	0.25
	single FD with 5% packing density coil	107.57	0.12	24.17	0.70
	single FD with 10% packing density coil	107.49	0.13	23.53	0.72
90°	pre-treated	136.49	0.23	1.84	3.73
	single FD	129.49	0.20	12.50	0.69
	overlapped FD with 50% overlapped rate	125.28	0.17	74.18	0.24
	overlapped FD with 25% overlapped rate	127.20	0.17	47.38	0.29
	single FD with 5% packing density coil	125.70	0.11	20.66	0.82
	single FD with 10% packing density coil	125.30	0.11	20.17	0.85
120°	pre-treated	148.05	0.20	1.72	4.35
	single FD	143.88	0.21	9.15	0.85
	overlapped FD with 50% overlapped rate	140.23	0.16	47.90	0.30
	overlapped FD with 25% overlapped rate	140.61	0.16	31.05	0.40
	single FD with 5% packing density coil	142.61	0.12	15.67	0.99
	single FD with 10% packing density coil	141.66	0.12	15.39	1.02
180°	pre-treated	187.65	0.26	1.09	5.06
	single FD	175.99	0.25	4.39	1.33
	overlapped FD with 50% overlapped rate	170.08	0.18	24.14	0.47
	overlapped FD with 25% overlapped rate	171.58	0.19	13.23	0.70
	single FD with 5% packing density coil	172.92	0.14	7.50	1.37
	single FD with 10% packing density coil	172.05	0.14	7.47	1.40
